# The Effect of Emotional Congruency on Multimedia Learning: An Eye Movement Study

**DOI:** 10.3390/bs15101330

**Published:** 2025-09-28

**Authors:** Lei Cui, Yuwei Zheng, Xiaoyu Song, Junfei Wang, Chengwei Liu, Ziyi Wang, Xiaofeng Zhang, Tingzhen Wang

**Affiliations:** 1 Faculty of Psychology, Shandong Normal University, Jinan 250358, China; cuilei@sdnu.edu.cn (L.C.); 19560851835@163.com (J.W.); lcw010704@163.com (C.L.); wzy13561590167@163.com (Z.W.); 2 Shandong Provincial Key Laboratory of Brain Science and Mental Health, Jinan 250358, China; 3 School of Education and Psychology, University of Jinan, Jinan 250022, China; 15306613626@163.com; 4 College of Psychology, Liaoning Normal University, Dalian 116029, China; 19741165012@163.com; 5 College of Food and Pharmaceutical Sciences and Technology, Shandong Vocational Animal Science and Veterinary College, Weifang 261061, China; zxfsssss@163.com

**Keywords:** multimedia learning, emotional design, emotional congruency, eye movement

## Abstract

This study employed eye-tracking technology to examine the emotional congruency effect of multimedia learning. The emotional congruency refers to the congruency of emotional valence between learning material and the emotional design in multimedia learning. With the internal emotional design, conducted positive and negative emotional designs for both positive and negative learning materials, thereby manipulating the emotional congruency between the emotional valence of learning material and the emotional design. Experiment 1 adopted positive learning materials and Experiment 2 adopted negative learning materials. The results showed that the congruent condition exhibited superior learning outcomes and eye-tracking performance compared to the incongruent condition, no matter whether the learning materials were emotionally positive or negative, and provided empirical evidence for the Cognitive-Affective Theory of Multimedia Learning and the emotional congruency effect. However, this results pattern did not appear regarding subjective perception. The emotional congruency effect and its implications to educational practice were discussed.

## 1. Introduction

Multimedia learning refers to the process of acquiring knowledge in the environment with texts, pictures and even videos (Mayer & Moreno, 2003). The multimedia environment can activate learners’ interest and motivation through multi-channel information presentation, thereby promoting the occurrence of meaningful learning more effectively (Mayer, 2014). Early research on multimedia learning focused on cognitive factors and proposed Cognitive Load Theory (CLT; Chandler, 1991) and Cognitive Theory of Multimedia Learning (CTML; Mayer et al., 2001). With the deepening of research on multimedia learning recently, researchers have found that emotional factors also play important role in multimedia learning, which means learners’ motivation, emotion and other subjective factors could affect learning effects (Wong & Adesope, 2021). Therefore, the exploration of factors that affect multimedia learning has gradually expanded from the initial single cognitive perspective to the systematic investigation of cognitive and emotional interaction.

The study of Moreno (2006) is the first one that had integrated the cognitive and emotional dimensions in multimedia learning study and developed the theory of Cognitive-Affective Theory of Learning with Media (CATLM), which is based on the cognitive theory of multimedia learning and integrates the emotional factors with the cognitive processing. Emotion includes two independent dimensions, valence and arousal (Russel, 1980; Stadthagen-Gonzalez et al., 2017). In this study, the emotion factor is the emotional valence dimension, which refers to the positive or negative experience of emotion. The theory proposes three theoretical hypotheses. Firstly, the emotional mediation hypothesis supposes that learners’ emotion and motivation affect the investment of cognitive resources, thereby influencing the effect of multimedia learning. Secondly, the metacognitive mediation hypothesis suggests that metacognitive resources exert an important effect on the learning process and outcomes by modulating learners’ cognitive processing and emotional states. Thirdly, the individual differences hypothesis states that learners’ differences, such as prior knowledge, cognitive style and ability, all have significant impact on multimedia learning process and outcomes. The CATLM has been widely confirmed by subsequent studies (Huang et al., 2022; Plass & Hovey, 2021; Park et al., 2014; Zhu et al., 2025; Zhao & Mayer, 2023, 2025).

According to the hypotheses of CATLM (Moreno, 2006), both emotional and cognitive factors would affect learning performance hierarchically. The CATLM posits that emotional factors might affect learning through their modulation on cognitive processes primarily through two mechanisms: (1) regulating cognitive engagement: positive emotions enhance learners’ motivation, making them more willing to invest necessary cognitive resources in processing learning materials. (2) Directing cognitive resources: emotions guide attention. Emotions such as interest and curiosity can direct attention toward learning content, whereas emotions like anxiety and boredom may divert attention to irrelevant matters or self-concerns, thereby wasting cognitive resources. Thus, in this study, we examined cognitive factors of intrinsic motivation, mental load, perceived difficulty, attention allocation and information integration, to explore through the cognitive mechanism which the emotional factor affects multimedia learning.

In recent years, with the guidance of the CATLM, researchers have paid attention to how the emotional design affects learners’ learning engagement and learning outcomes (Linnenbrink-Garcia & Pekrun, 2011; Mutlu-Bayraktar, 2024). The concept of emotional design was first proposed by Um et al. (2012) and means the use of various design features with the goal to impact learners’ emotions to enhance learning (Navratil et al., 2018; Plass & Kaplan, 2016; Park et al., 2015). These design features should not add additional information to the learning materials but should comprise information that can influence learners’ emotional states, such as specific color combinations, anthropomorphism or baby face characteristics (Navratil et al., 2018; Plass et al., 2014; Um et al., 2012; Xu, 2017). Previous studies confirmed that saturated and bright warm colors with anthropomorphic baby smiling faces could induce positive emotions (Plass et al., 2014; Um et al., 2012), while low saturated and dark purple color combinations with anthropomorphic crying faces could induce negative emotion (Navratil et al., 2018; Xu, 2017). With the implementation of two contrasting emotional valence conditions, the emotional design induced learner’s positive and negative emotion respectively, aiming to explore how learner’s emotional state affects learning. Existing findings can be summarized into three groups. The first one is the emotional facilitation hypothesis, which holds that positive emotional design can promote learning. In detail, positive emotional design can reduce learners’ perceived difficulty (Plass et al., 2014), enhance learners’ motivation (Xue et al., 2015) and increase their mental effort (Um et al., 2012), thereby improving learning outcomes (Brom et al., 2018; Mayer et al., 2014; Plass & Hovey, 2021; Wong & Adesope, 2021). The second one is the emotional inhibition hypothesis, which suggests that positive emotional design can impair learning. For example, cognitive load theory holds that emotion is a kind of extraneous cognitive load, which occupies individuals’ limited cognitive resources, and this occupation of resources hinders learners’ in-depth learning (Leppink & Duvivier, 2016; Plass & Kaplan, 2016). The third one is the null effect hypothesis, which proposes that emotional design has no impact on learning (Heidig et al., 2015; Park et al., 2015).

These theoretical hypotheses have been supported by empirical research selectively. Mutlu-Bayraktar (2024) conducted a meta-analysis, where the results of 16 studies showed that positive emotional design promoted learning outcomes whilst 10 studies showed null effect. Notably, the above studies mainly focus on the impact of positive emotional design on multimedia learning; however, some studies further examined the impact of both positive and negative emotional designs on multimedia learning (Chung & Cheon, 2020; Liew et al., 2022; Navratil et al., 2018). Liew et al. (2022) found that the positive emotion group had better learning outcomes compared with the negative emotion group and the neutral group. Navratil et al. (2018) adopted the same emotional design as Um et al. (2012), but found that the negative emotion group achieved better learning outcomes than the positive and neutral groups, which was contrary to the previous theoretical hypothesis. To sum up, the impact of emotional design on multimedia learning outcomes remains controversial.

The above analysis shows discrepancies regarding the impact of learners’ emotion on the learning motivation and outcomes; thus, a unified conclusion has not yet been reached. As mentioned, Navratil et al. (2018) adopted the same emotional design as Um et al. (2012); they assumed that positive emotional design would be associated with a higher motivation to accomplish the learning activity. Moreover, an interaction was expected: learners learning with a positive emotional design should profit from learning whereas learners learning with a negative emotional design would not profit from this strategy to the same extent. But they found that the negative emotion group achieved better learning outcomes than the positive and neutral groups. They explained that the content of the “immune theme” learning materials in the study was negative emotional valence, when the emotional design was also negative, the congruent emotional cues reduce the cognitive load of the learners, thereby improved the learning performance. Analyzing the learning materials used in previous studies, researchers mainly focused on the difficulty or the topic of learning materials, but less pay attention to the emotional valence (i.e., positive or negative). Therefore, whether the emotional congruency between the emotional valence of learning materials and the emotion induced by emotional design is the possible reason for the inconsistent researching conclusions in multimedia learning, it is a question worthy of further exploring, which is the main purpose of present study.

Bower (1981) proposed that when individuals are in a certain emotional state, they tend to select and process information that is congruent with current emotion, and exhibit a kind of emotional priming effect, which is called the emotional congruency effect. According to Bower (1981) and Navratil et al. (2018), the emotional congruency was defined as the congruency of emotional valence between learning material and the emotional design in multimedia learning. Bower (1978) induced participants’ positive or negative emotions under a hypnotic environment, and then asked them to read stories with positive or negative emotional valence, respectively. The results showed better recall grades for stories that have congruent emotion with their own emotional state. Beege et al. (2018) induced learners’ positive or neutral emotion by presenting pictures with positive or neutral facial expressions, respectively, and then asked the participants to learn multimedia learning videos with positive or neutral emotion. The results showed that the learning outcomes of the emotional congruency group were better than the emotional incongruent group. Mei and Lu (2014) conducted a study on classroom learning. In the study, learners’ positive and negative emotions were first induced by watching positive and negative movie clips, and then they were asked to participate in classroom learning with positive and negative emotional materials. They found that the emotional congruent group was better than the emotional incongruency group in terms of learning interest, learning motivation and self-efficacy. The above studies support the emotional congruency effect, but they all used external emotion induction to induce learners’ corresponding emotional states.

External emotional induction refers to applying a series of emotional induction methods in order to induce their positive or negative emotion before the experiment, for example, watching emotional movie clips, reading emotional articles, listening to music combined with autobiographical memory, etc. (Gong et al., 2017; Knörzer et al., 2016; Park et al., 2015; Plass et al., 2014; Um et al., 2012). However, external emotional induction has some shortcomings. For example, the time for external emotional induction is relatively long and is even longer than the learning time. Moreover, it is difficult to guarantee that the induced emotion is maintained for the entire learning process. Therefore, the ecological validity of external emotional induction is low (Um et al., 2012). Fortunately, internal emotional induction can solve the above problems. Internal emotional induction refers to the way of adding designed visual elements (e.g., pictures, colors, etc.) in learning materials that can elicit learners’ emotions and promote their learning (Plass et al., 2014). Internal emotional induction is induced by the learning materials themselves, which can maintain the entire learning process and has high ecological validity compared with external emotional induction (Peng et al., 2021). Whether internal emotion induction can provide further evidence to the emotional congruency effect remains unsolved. Therefore, the present study will explore the impact of emotional congruency on multimedia learning with internal emotional induction.

Recently, eye-tracking technology has been more and more used in multimedia learning studies. Compared with traditional cognitive measurement methods, eye-tracking technology can objectively record learners’ eye movement data during multimedia learning, thereby reflecting learners’ real-time cognitive processing and examining visual attentional distribution patterns and information processing without causing interference to learners’ learning processes (H. Li & Zheng, 2024). Eye-tracking technology can objectively quantify learners’ attention allocation patterns to learning content and reveal learners’ cognitive processing (Peng et al., 2021; Sondermann et al., 2024); therefore, eye-tracking technology is particularly well-suited for studying multimedia learning (Hyönä et al., 2021; Liu & Cui, 2025; Rayner, 1998; Stark et al., 2024). Fixation duration and fixation count can be used as a reflection of the cognitive processing during multimedia learning. A longer fixation duration and higher fixation count mean more cognitive processing is directed toward the information (Koçoğlu et al., 2021; W. Li et al., 2024; Z. L. Pi et al., 2023). The number of transitions between text and picture information reflects the integrated processing of information and more transitions mean the information integration is more difficult (Genc Aksaray & Ozcelik, 2023; Stark et al., 2018; Wang et al., 2023). Thus, this study combines objective (i.e., eye-tracking indicators) and subjective (intrinsic motivation, mental load, perceived difficulty) methods to measure cognitive processes, with the hope that they could complement each other to reveal the underlying mechanisms through which the emotional factor affects multimedia learning. In particular, with the eye-tracking indicators, we might explore the link between objectivity and subjectivity, e.g., the link between emotional state and subjective difficulty or mental effort, and even learning performance. The attentional resource and information integration, which are measured by eye-tracking, is hypothesized to be the core cognitive mechanisms that modulate multimedia learning.

To sum up, this study employed eye-tracking technology to explore the impact of emotional congruency between the emotional valence of learning material and the emotional design on multimedia learning. Experiment 1 adopted positive learning materials meanwhile positive (congruent) and negative (incongruent) emotional designs were conducted. Experiment 2 adopted negative learning materials meanwhile negative (congruent) and positive (incongruent) emotional designs were conducted. Based on the theory of emotional congruency effect (Bower, 1981), the hypotheses of this study were as follows:

(1) In terms of learning outcomes, learners in the congruent condition would perform better than those in the incongruent condition on both retention and transfer tests.
(2) Regarding subjective perception, learners in the congruent condition would show higher intrinsic motivation, lower perceived difficulty and lower mental effort than those in the incongruent condition.
(3) In terms of eye movement measures, fixation duration, fixation count and the number of transitions on the learning material would be less in the congruent condition than in the incongruent condition.

## 2. Experiment 1: The Impact of Emotional Congruency on Positive Learning Material

### 2.1. Methods

#### 2.1.1. Participants

A priori power analysis was conducted with G*Power 3.1.9. Based on related research from Zhang et al. (2024), the effect size for Transfer was 0.89, a statistical power of 0.80 and a significance level (α) of 0.05, then a sample size of at least 34 was needed. According to this simple size, a total of 40 participants were recruited. The analyzing power was validated after the experiment using the actual data. This analysis revealed an observed effect size (Cohen’s *d*) of 0.93, with a significance level (*α*) of 0.05 and a statistical power of 0.82, which is greater than the desired statistical power of 0.80, indicating that the experimental results have sufficient explanatory power.

The 40 participants (30 females) were recruited via posters from Shandong Normal University in China; they were aged 18–24 years old (*M* = 21.73, *SD* = 1.68). All participants were unfamiliar with the topic of learning. The participants come from different majors, including engineering, geographic information science, educational technology and so on. Each participant received payment after completing the experiment. The local ethics committee approved the study protocol.

#### 2.1.2. Experimental Design

The experiment adopted positive learning materials meanwhile positive and negative emotional designs were conducted, thereby resulting in the congruent condition (positive learning material combined with positive emotional design) and incongruent condition (positive learning material combined with negative emotional design). Therefore, the experiment was a single-factor between-subjects design. The independent variable was the emotional congruency condition (congruent vs. incongruent). The 40 participants were randomly assigned to each experimental condition, half and half. The dependent variables were learning outcomes (retention test and transfer test), subjective perception (intrinsic motivation, mental effort and perceived difficulty) and eye movement indices including the fixation duration of areas of interest (AOIs), fixation count of AOIs and the number of transitions between text and picture areas.

#### 2.1.3. Learning Materials

A multimedia learning environment on “emotional promotion effect” designed by Feng (2020) served as learning material. It incorporated text, pictures and animations accompanied by narration. The learning was self-paced and did not allowed learners to replay, but the maximal duration of the per slide was 1 min. The learning content was presented in seven chapters with a total duration of 7 min.

**Figure 1:**
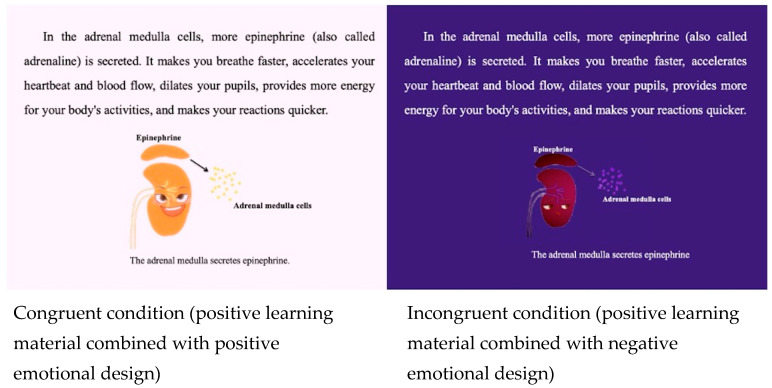
Positive learning materials combined with positive emotional design (**left** side, congruent condition) and negative emotional design (**right** side, incongruent condition).

For the two emotional designs, the positive emotional design used saturated and analogous bright warm color combinations including anthropomorphic baby smiling faces (Plass et al., 2014; Um et al., 2012); the negative emotional design used low saturated and analogous dark purple color combinations including anthropomorphic crying faces (Navratil et al., 2018; Xu, 2017). The emotional impact of these emotional designs has been established in previous empirical research and they allow for the design of a variant of the treatments without adding any new learning content and thus avoid confounding. The differences of the two emotional designs only involved the color and shape of the anthropomorphic faces, the positive and the negative design materials both had the same amount of learning content, user control options, duration and applied the same multimedia learning design principles (Mayer, 2005). Figure 1 shows the positive learning material (In the formal experiment, the learning materials were in Chinese.).

Before the formal experiment, 15 undergraduates from Shandong Normal University were recruited to rate the emotional valence of the internal emotional design on a 9-point Likert scale ranging from 1 (not at all positive/negative) to 9 (extremely positive/negative). A repeated measures *t*-test was performed on the ratings. The results showed that in terms of positive emotion, the score of positive internal emotional design (*M* = 7.00, *SD* = 0.53) was significantly higher than that of negative internal emotional design (*M* = 2.72, *SD* = 0.79), *t* (28) = 17.40, *p* < 0.01. In terms of negative emotion, the score of negative internal emotional design (*M* = 7.10, *SD* = 0.57) was significantly higher than that of positive internal emotional design (*M* = 2.60, *SD* = 0.58), *t* (28) = 21.18, *p* < 0.01. These results confirmed that the emotional designs could induce the intended positive and negative emotional states.

In addition, before the experiment another 15 undergraduates who did not take part in the formal experiment and the emotional design pilot test were recruited to rate the emotional valence of the learning text using the Positive and Negative Affect Scale (PANAS; Qiu et al., 2008). For the positive learning material, the positive emotion score (*M* = 30.43, *SD* = 6.93) was significantly higher than the negative emotion score (*M* = 14.86, *SD* = 4.88), *t* (28) = 3.60, *p* < 0.01. These results indicate that the positive learning materials successfully induced the positive emotions.

#### 2.1.4. Assessment Instruments

Prior knowledge test: Prior knowledge was assessed using a four-item self-report checklist. Participants received one point for each item that they reported, which span from “I know the function of the pituitary gland” to “I know the functions of emotions”. The total score for each participant was obtained by adding points from all four items. And the internal consistency reliability (Cronbach’s *α*) was 0.71.

Positive and negative affect scale: The positive and negative affect scale (PANAS; Watson et al., 1988) was used, in which nine emotion items were used to measure participants’ positive affect (PA) and nine emotion items were used to measure their negative affect (NA). Before and after the multimedia learning, participants rated their emotions on a 7-point Likert-type scale ranging from 1 (not at all) to 7 (very strong). The internal consistency reliability (Cronbach’s *α*) of PA and NA were 0.85 and 0.84, respectively.

Learning performance tests: The retention and transfer tests were administered. The retention test measured learners’ understanding of the key concepts of the materials and was composed of six single-choice questions with maybe four possible answers (e.g., “Which of the following statements is incorrect regarding positive emotions?” Cronbach’s *α* = 0.85). Learners received one point for each correctly answered item. The transfer test contained two open questions (e.g., “How to promote the improvement of cognitive ability”). Participants received up to twenty points for answering these two questions; the scores were rated by two independent raters who rigorously trained graduate students (ICC(2, 2) = 0.96) and they could get assistance from two expert teachers in the relevant field. And the internal consistency reliability (Cronbach’s *α*) was 0.96.

Intrinsic motivation scale: Intrinsic motivation is defined as interest and enjoyment arising from the learning task itself (Ryan & Deci, 2000). A learner’s intrinsic motivation was measured using a self-report instrument consisting of an eight-item questionnaire (e.g., It is enjoyable) with 7-point Likert-type scale (1 strongly disagree, 7 strongly agree) developed by Isen and Reeve (2005). Participants were asked to rate how interesting and enjoyable they felt; 1–7 points were assigned for each item, and a participant’s total score obtained by adding responses to the eight items. And the internal consistency reliability (Cronbach’s *α*) was 0.93.

Cognitive load scale: To measure the cognitive load experienced by learners, participants completed a 9-point Likert-type Cognitive Load Subjective Experience Questionnaire (e.g., “How much mental effort did you invest in studying the previous material?” Gong et al., 2017; Um et al., 2012). Participants also completed a 7-point Likert-type survey on their perceptions of task difficulty (e.g., “How easy or difficult was the material to understand?” Park et al., 2015).

#### 2.1.5. Eye Movement Indices

The experiment employed the Eye-link 1000 Plus eye tracker, which is made by SR Research Company of Canada, with a sampling rate 1000 Hz. The learning materials were presented on a 21-inch CRT display with a resolution of 1024 × 768 pixels. The participant’s eyes were kept 60 cm from the monitor.

**Figure 2:**
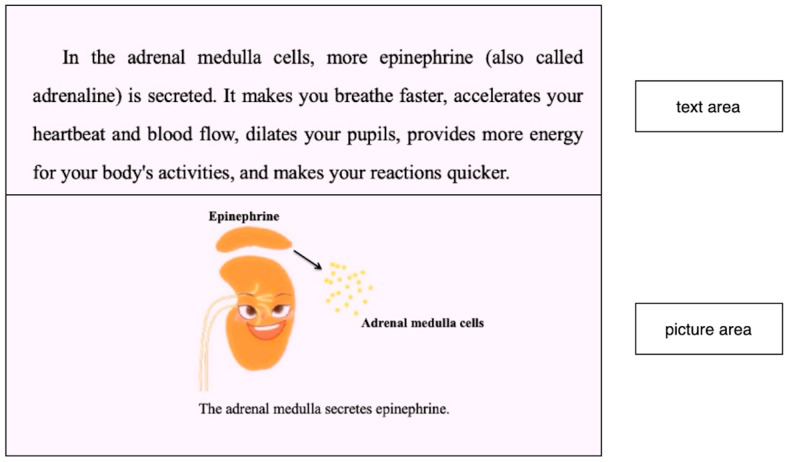
The two areas of interest (AOIs) in learning for data recording and analysis. Upper for text area and lower for picture area.

Two areas of interest (AOIs) were designed: text and picture areas (as shown in Figure 2). The text and picture were in the upper and lower positions, respectively. According to Manor and Gordon (2003), a fixation duration of 100 ms is considered the boundary between fixation and other eye movement activities. Therefore, fixations less than duration of 100 ms were excluded from the eye movement data analyses. We selected three eye-tracking measures for analysis: fixation duration, fixation count and the number of transitions. Fixation duration reflects the total amount of time fixated on the AOI. Fixation count reflects the total number of fixations on the AOI. The number of transitions reflects the number of times that the learner’s fixation shifts between the different AOIs.

#### 2.1.6. Experimental Procedure

**Figure 3:**
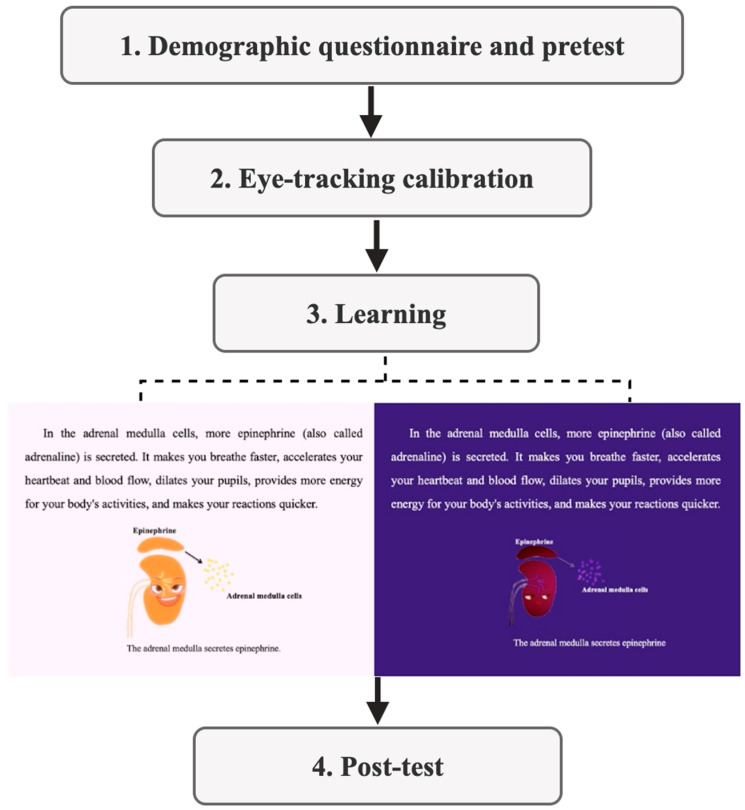
Experimental procedure, which include four stages lasting for about 18 min in total.

The experimental procedure is illustrated in Figure 3, which included four stages:

Firstly, participants completed demographic information, followed by a prior knowledge test and a pre-test of emotion assessment.

Secondly, participants were invited into the eye-tracking laboratory for seat adjustment. They then received experimental instructions. Subsequently, participants were seated in front of the eye tracker with their chin placed on a chin rest and a 9-point calibration was performed to ensure accurate recording of eye movement trajectories.

Thirdly, participants were randomly assigned to one of the two experimental groups to learn corresponding materials, during which their eye movements were tracked.

Finally, immediately after learning, participants completed an emotion assessment, followed by an intrinsic motivation scale, a cognitive load scale and learning performance test. The entire experiment lasted approximately 18 min. Upon completion of the experiment, participants received corresponding compensation.

### 2.2. Results

#### 2.2.1. Emotional Induction

For participants’ initial emotional state, as shown in Table 1, an independent samples *t*-test on the pre-test found that there was no significant difference on positive emotion between the two conditions, *t*(38) = −0.39, *p* = 0.35, *d* = −0.12, and there was no significant difference on negative emotion between the two conditions, *t*(38) = 1.71, *p* = 0.05, *d* = 0.53.

For emotional induction results, the paired-samples *t*-tests were conducted to compare post-test with pre-test in two experimental conditions. For the congruent condition, the positive emotion increased significantly, *t*(19) = −1.98, *p* = 0.03, *d* = −0.44, *r* = 0.77, and the negative emotion decreased significantly, *t*(19) = 2.56, *p* = 0.01, *d* = 0.57, *r* = 0.72. For the incongruent condition, the positive emotion decreased significantly, *t*(19) = 1.902, *p* = 0.03, *d* = 0.43, *r* = 0.87, and the negative emotion did not change, *t*(19) = −0.380, *p* = 0.35, *d* = −0.09, *r* = 0.44.

#### 2.2.2. Learning Outcomes

To ensure that the differences in the experiment results were not caused by differences in participants’ prior knowledge and emotional states, we first conducted independent sample *t*-tests and found the scores of the prior knowledge test across the two conditions. The results showed no significant differences between the congruent condition (*M* =12.50, *SD* = 3.72) and incongruent condition (*M* = 13.40, *SD* = 2.09), *t*(38) = −0.94, *p* = 0.18, *d* = −0.30.

Table 2 shows the results of independent sample *t*-tests on learning outcomes. For the retention performance, the congruent condition was significantly higher than that in the incongruent condition, *t*(38) = 2.15, *p* = 0.02, *d* = 0.67. For the transfer performance, the congruent condition was significantly higher than that in the incongruent condition, *t*(38) = 2.93, *p* = 0.003, *d* = 0.93.

#### 2.2.3. Subjective Perception

For the results of three subjective perceptions, as shown in Table 3, based on independent sample *t*-tests, there was no significant difference on intrinsic motivation between the two conditions, *t*(38) = 0.94, *p* = 0.18, *d* = 0.30; there was a significant difference on the perceived difficulty between the two conditions, *t*(38) = −1.77, *p* = 0.04, *d* = −0.56; and there was no significant difference on mental effort between the two conditions, *t*(38) = −0.21, *p* = 0.42, *d* = −0.07.

#### 2.2.4. Eye Movement Indices

First, the eye movement data were screened with the EyeLink Dataviewer data software 5.1.1 and the data with a fixation duration lower than 100 ms or exceeding 2.5 standard deviations were deleted. The deleted data account for 6.1% of the total data. The eye movement data were processed using R (version 3.3.1 R: Development Core Team) and LME4 data packet (version 1.1–12: Bates et al., 2015) of the linear mixed model.

The results of the R-analysis (see Table 4) showed that there was a significant difference on the fixation duration in the text area between the two conditions (*b* = 0.25, *SE* = 0.12, *t* = 2.04, *p* < 0.05) and the fixation duration in the congruent condition was significantly shorter than that in the incongruent condition. In terms of fixation count in the text area, there was a significant difference between the two conditions (*b* = 0.25, *SE* = 0.11, *t* = 2.31, *p* = 0.02) and the fixation count in the congruent condition was significantly less than those that in the incongruent condition.

Additionally, for the picture area, there was a marginally significant difference on fixation duration between the two conditions (*b* = 0.45, *SE* = 0.23, *t* = 1.96, *p* = 0.06) and the fixation duration of the congruent condition was shorter than that of the incongruent condition. In terms of fixation duration in the picture area, there was a marginally significant difference between the two conditions (*b* = 0.41, *SE* = 0.21, *t* = 1.93, *p* = 0.06) and the fixation duration in the congruent condition was shorter than that in the incongruent condition. In terms of the number of transitions between the text area and the picture area, there was a significant difference between the two conditions (*b* = 0.24, *SE* = 0.10, *t* = 2.42, *p* = 0.02) and the number of transitions in the congruent condition was significantly less than that in the incongruent condition.

### 2.3. Discussion

The experiment employed positive learning materials to explore the impact of emotional congruency between the emotional valence of learning material and the emotional design on multimedia learning. Referring to existing research findings (Navratil et al., 2018; Plass et al., 2014; Um et al., 2012; Xu, 2017), we conducted a positive internal emotional design using high-saturation warm colors paired with anthropomorphic smile icons to induce positive emotion; in contrast, the negative internal emotional design using low-saturation cool colors with anthropomorphic crying icons to induce negative emotion. Post-learning emotional assessments showed that the congruent condition demonstrated increased positive emotion and decreased negative emotion compared to pre-learning baselines, indicating that positive emotional induction was effective. Conversely, the incongruent condition (positive learning material with negative emotional design) exhibited decreased positive emotion, indicating that negative emotional induction was also effective. But for negative emotion scores, there was no significant difference. This null difference may stem from the mutual cancellation of emotional effects between the positive learning material and the negative emotional design and it also indicates the negative emotional design was effective. For learning outcomes, learners in the congruent condition performed better than those in the incongruent condition on both retention and transfer tests, indicating the congruent condition between emotional valence of the learning material and emotional design is more conducive to the learning outcomes. Eye-tracking measures further revealed a significantly shorter fixation duration and fewer fixation counts in the text areas for the emotional congruent condition compared to the emotional incongruent condition. The same numerical trend emerged in the picture area. Similarly, the number of transitions is fewer between the text, and picture Areas of Interest (AOIs) were observed in the congruent condition than the incongruent condition. Therefore, these results consistently supported the emotional congruency effect.

However, it is important to note that these results can also be interpreted using the positivity principle, which posits that positive emotion can promote participants’ learning motivation, thereby improving learning outcomes (Horovitz & Mayer, 2021; Z. Pi et al., 2022; Z. L. Pi et al., 2023). In the congruent condition of this study, emotional design induced learners’ positive emotion; whereas in the incongruent condition, emotional design induced learners’ negative emotion. That means that the congruent condition accompanies with positive emotional design; nevertheless, the incongruent condition accompanies negative emotional design. The two factors mixed together, therefore, the better learning outcomes in the congruent condition compared with incongruent condition might also be attributed to positive emotional design, rather than solely to the emotional congruency effect. To rule out this potential confounding, in Experiment 2, we employed negative learning material and designed emotional designs that were negative and positive for the congruent and incongruent conditions, respectively, to further investigate the impact of emotional congruency on multimedia learning. If we could find the better learning outcomes for the congruent condition than the incongruent condition, then this could only be attributed to the emotional congruency effect.

## 3. Experiment 2: The Impact of Emotional Congruency on Negative Learning Material

### 3.1. Methods

#### 3.1.1. Participants

The 40 participants (32 females) who did not take part in Experiment 1 were recruited via posters from a university in China; they were aged 18–24 years old (*M* = 21.57, *SD* = 1.96). All participants were unfamiliar with the topic of learning. The participants come from different majors, including engineering, geographic information science, educational technology and so on. Each participant received payment after completing the experiment. The local ethics committee approved the study protocol.

#### 3.1.2. Experimental Design

The experiment adopted negative learning materials, while positive and negative emotional designs were conducted, thereby resulting in the congruent condition (negative learning material combined with negative emotional design) and incongruent condition (negative learning material combined with positive emotional design). Therefore, the experiment was a single-factor between-subject design. The independent variable was the emotional congruency condition (congruent vs. incongruent). The 40 participants were randomly assigned to each experimental condition, half and half. The dependent variable and control variable were the same as in Experiment 1.

#### 3.1.3. Learning Materials

**Figure 4:**
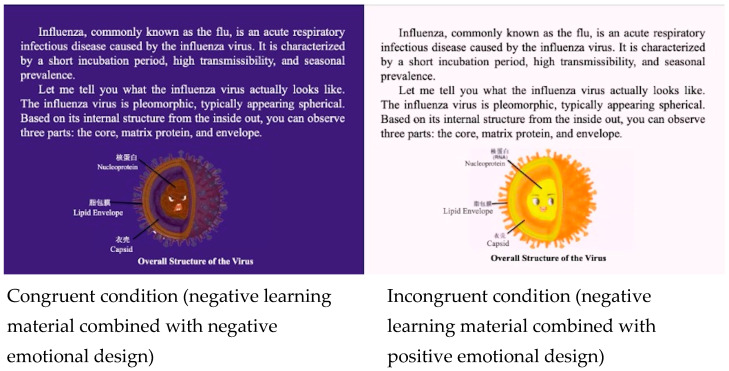
Negative learning materials combined with negative emotional design (**left** side, congruent condition) and positive emotional design (**right** side, incongruent condition).

A multimedia learning environment on “The process of influenza” designed by Feng (2020) served as learning material. It incorporated text, pictures and animations accompanied by narration. The learning was self-paced and did not allowed learners to replay, but the maximal duration of the per slide was 1 min. The learning content was presented in ten chapters with a total duration of 10 min. The positive and negative internal emotional design in this study was the same as that of Experiment 1. The negative learning materials are shown in Figure 4 (In the formal experiment, the learning materials were in Chinese.).

Before the formal experiment, 15 undergraduates from Shandong Normal University were recruited to rate the emotional valence of the internal emotional design on 9-point Likert scale ranging from 1 (not at all positive/negative) to 9 (extremely positive/negative). A repeated measures *t*-test was performed on the ratings. The results showed that in terms of positive emotion, the score of positive internal emotional design (*M* = 7.01, *SD* = 0.57) was significantly higher than that of negative internal emotional design (*M* = 6.87, *SD* = 0.47), *t*(28) = 17.19, *p* < 0.01. In terms of negative emotion, the score of negative internal emotional design (*M* = 6.87, *SD* = 0.47) was significantly higher than that of positive internal emotional design (*M* = 2.48, *SD* = 0.54), *t*(28) = 23.79, *p* < 0.01. These results confirmed that the emotional designs could induce the intended positive and negative emotional states.

In addition, before the experiment another 15 undergraduates who did not take part in the formal experiment and the emotional design pilot test were recruited to rate the emotional valence of the learning text using the Positive and Negative Affect Scale (PANAS; Qiu et al., 2008). For the negative learning material, the negative emotion score (*M* = 19.75, *SD* = 5.93) was significantly higher than the positive emotion score (*M* = 11.50, *SD* = 5.47), *t*(28) = 2.31, *p* < 0.05. These results indicate that the positive learning materials successfully induced the negative emotion.

#### 3.1.4. Assessment Instruments

Assessment instruments (PANAS: Cronbach’s *α* of PA = 0.88, Cronbach’s *α* of NA = 0.73; intrinsic motivation scale: Cronbach’s *α* = 0.87; cognitive load scale: Cronbach’s *α* = 0.78) were the same as Experiment 1, except for the prior knowledge test and the learning performance tests (i.e., retention and transfer tests).

Prior knowledge test: Prior knowledge was assessed using a four-item self-report checklist. Participants received one point for each item that they reported, which span from “I know what an antigen is” to “I know how influenza spreads”. The total score for each participant was obtained by adding points from all four items. And the internal consistency reliability (Cronbach’s *α*) was 0.85.

Learning performance tests: The retention and transfer tests were administered. The retention test measured learners’ understanding of key concepts of the materials and was composed of six single-choice questions with maybe four possible answers (e.g., “Which of the following is NOT a characteristic of influenza?” Cronbach’s *α* = 0.78). Learners received one point for each correctly answered item. The transfer test contained two open questions (e.g., “How to prevent influenza”). Participants received up to twenty points for answering these two questions; the scores were rated by two independent raters as Experiment 1 (ICC(2, 2) = 0.81). And the internal consistency reliability (Cronbach’s *α*) was 0.86.

#### 3.1.5. Eye Movement Indices

Eye movement indices were the same as Experiment 1.

#### 3.1.6. Experimental Procedure

Experimental procedure was the same as Experiment 1.

### 3.2. Results

#### 3.2.1. Emotional Induction

As shown in Table 5, for initial emotional state, independent sample *t*-tests on the pre-test found that there was no significant difference in positive emotion, *t*(38) = 0.68, *p* = 0.25, *d* = 0.21, and there was no significant difference in negative emotion, *t*(38) = −1.55, *p* = 0.06, *d* = −0.55.

For emotional induction results, the paired-sample *t*-test were conducted to compare post-test with pre-test in two experimental conditions. For the congruent condition, the positive emotion decreased significantly, *t*(19) = 2.45, *p* = 0.01, *d* = 0.55, *r* = 0.72, and the negative emotion increased significantly, *t*(19) = −1.97, *p* = 0.03, *d* = −0.44, *r* = 0.63. For the incongruent condition, there was no significant difference in positive emotion, *t*(19) = 0.22, *p* = 0.41, *d* = 0.05, *r* = 0.38, and negative emotion, *t*(19) = 1.29, *p* = 0.11, *d* = 0.29, *r* = 0.68.

#### 3.2.2. Learning Outcomes

To ensure that the differences in the experiment results were not caused by differences in participants’ prior knowledge and emotional states, we first conducted independent sample *t*-tests and found the scores of the prior knowledge test across the two conditions. The results showed no significant differences between congruent condition (*M* = 13.50, *SD* = 3.55) and incongruent condition (*M* = 13.20, *SD* = 3.35), *t*(38) = 0.28, *p* = 0.39, *d* = 0.01.

Table 6 shows the results of learning outcomes. The independent sample *t*-tests found that there was a significant difference on retention performance, *t*(38) = 2.40, *p* = 0.01, *d* = 0.76. The retention performance in the congruent condition was significantly higher than the incongruent condition. In terms of transfer performance, the difference was significant, *t*(38) = 2.37, *p* = 0.01, *d* = 0.75. The transfer performance in the congruent condition was significantly higher than that in the incongruent condition.

#### 3.2.3. Subjective Perception

Independent sample *t*-tests were conducted on subjective perceptions. The results are presented in Table 7. In terms of intrinsic motivation, the results showed there was no significant difference between the two conditions, *t*(38) = −0.61, *p* = 0.27, *d* = −0.19. In terms of perceived difficulty, there was no significant difference between the two conditions, *t*(38) = 0.19, *p* = 0.43, *d* = 0.06. In terms of mental effort, there was no significant difference between the two conditions, *t*(38) = −0.95, *p* = 0.18, *d* = −0.30.

#### 3.2.4. Eye Movement Indices

The eye movement data were screened with the same standards as Experiment 1. The deleted data account for 6.19% of the total data. The eye movement data were processed by the R data packet (version 3.3.1 R: Development Core Team) and LME4 data packet (version 1.1–12: Bates et al., 2015) of the linear mixed model.

The results of the R-analysis (also see Table 8) showed that there was a significant difference between the two conditions on the fixation duration of the picture area (*b* = 0.38, *SE* = 0.12, *t* = 3.16, *p* < 0.01) and the fixation duration of the congruent condition was shorter than that of the incongruent condition. In terms of the fixation count in the picture area, there was a significant difference between the two conditions (*b* = 0.36, *SE* = 0.11, *t* = 3.19, *p* < 0.01) and the fixation count in the congruent condition was less than that in the incongruent condition. In terms of the number of transitions between the text area and the picture area, there was a significant difference between the two conditions (*b* = 0.24, *SE* = 0.08, *t* = 2.91, *p* < 0.01) and the number of transitions in the congruent condition was significantly less than that in the incongruent condition.

### 3.3. Discussion

This experiment employed negative learning materials to explore the emotional congruency effect between the emotional valence of learning material and the emotional design on multimedia learning. It adopted identical emotional design methods as Experiment 1 and pre-post emotional assessments confirmed that the emotional design was effective. The congruent condition (negative learning material with negative emotional design) showed a decreased positive emotion and an increased negative emotion after learning, demonstrating that negative emotional induction was effective. The incongruent condition (negative learning material with positive emotional design) showed there was no significant difference after learning. This null difference may stem from mutual cancellation of emotional effects between the negative learning material and the positive emotional design and it also indicates the positive emotional design was effective.

For learning outcomes, learners in the congruent condition performed better than the incongruent group on both retention and transfer tests. Specifically, when the emotional valence of learning material and the emotional design were congruent (i.e., were both negative), learners achieved superior learning outcomes, which supports the emotional congruency effect. The emotional congruency effect was further found in real-time learning process. Eye-tracking measures revealed significantly shorter fixation duration, fewer fixation counts and a reduced number of transitions between the Areas of Interest (AOIs) in the picture area for the congruent condition. This pattern suggests that the congruent condition was more conducive to learners’ learning, which confirmed the conclusions of Experiment 1.

## 4. General Discussion

This study employed eye-tracking technology to examine the impact of emotional congruency on multimedia learning. From the perspective of internal emotional design, we conducted positive and negative emotional designs for positive and negative learning materials, thereby manipulating the emotional congruency between the emotional valence of learning material and the emotional design. Results showed that the congruent condition exhibited superior learning outcomes and eye-tracking performance compared to the incongruent condition, providing empirical evidence for the Cognitive-Affective Theory of Multimedia Learning and the emotional congruency effect.

### 4.1. The Influence of Emotional Congruency on Multimedia Learning Outcomes

In the field of multimedia learning, the retention test is typically employed to assess learners’ memories of learning materials (Mayer, 2005) and the transfer test is employed to evaluate learners’ abilities to apply acquired knowledge in solving problems (Atkinson, 2002). Regarding learning outcomes, the congruent condition performed better than the incongruent condition on both retention and transfer tests. Firstly, these findings support the emotional mediation hypothesis of the Cognitive-Affective Theory of Multimedia Learning (Moreno, 2006), indicating that learners’ emotional states influence their cognitive resource allocation, thereby affecting multimedia learning outcomes. This was also consistent with the meta-analysis results of Brom et al. (2018), which indicated that incorporating anthropomorphism and pleasant colors into multimedia learning materials was an effective emotional design and can positively influenced retention, comprehension and transfer performance. The results were also consistent with some of the findings of Mutlu-Bayraktar (2024). More importantly, these results further validate the emotional congruency effect (Bower, 1981), which is when people are in a certain emotional state, they tend to select and process the information that is consistent with their emotion. In Experiment 1, with a positive emotional design, learners benefited to process positive learning material; in Experiment 2, with a negative emotional design, learners benefited to process negative learning material.

The Affect Infusion Model (AIM; Forgas, 1995; Forgas & George, 2001) offers a theoretical framework for understanding the emotional congruent effect. AIM posits that people’s emotional state systematically modulates the depth and the strategy of information processing, thereby indirectly and systematically influencing cognitive processing. Specifically, the influence of emotion on cognitive processing depends on the depth, openness and duration of the cognitive task. As the task becomes more complex, the infusion of emotion grows, leading individuals’ cognition to align more closely with their emotional state. The emotionally incongruent design may function as an extraneous cognitive load, interfering with the selection and organization of information. This may require attentional resources to be split between semantic processing and emotional regulation, wherein incongruent emotional cues as a sort of affective interference disrupt processing flow in working memory. Correspondingly, under the emotionally congruent design, there is no need for learners to split working memory resources to reconcile emotional conflict, allowing a thorough allocation to cognitive processing. This mechanism reveals an emotion’s role as a “cognitive catalyst” that optimizes knowledge integration.

As mentioned earlier in Experiment 1, although the congruent condition performed better than the incongruent condition on learning outcomes, these results could alternatively be explained by the positivity principle (Horovitz & Mayer, 2021). In the congruent condition, emotional design induced positive emotion, whereas in the incongruent condition, emotional design induced negative emotion. According to the positivity principle, positive emotion improved learners’ motivation, thereby improving learning outcomes. Consequently, we conducted Experiment 2, which employed negative learning material paired with negative (congruent) and positive (incongruent) emotional designs. Again, results revealed better retention and transfer tests in the congruent condition (negative emotional design with negative learning material) compared with the incongruent condition (positive emotional design with negative learning material). The results can be explained by the emotional congruency effect rather than the positivity principle. Considering the two experiments together, the results showed clearly that the emotional congruency effect has a pronounced advantage. Consequently, the impact of the emotional congruency effect should be considered firstly when a designer conducts emotional designs on multimedia learning.

The emotional congruency effect can explain inconsistent findings in prior research. Analyzing the learning materials used in previous studies, researchers mainly focused on the difficulty of the learning material, but did not pay attention to the emotional valence (i.e., positive or negative) of the learning material. The emotional incongruency between the emotional valence of learning material and the emotion induced by emotional design may lead to different, or even diametrically opposite, learning outcomes. This study provides practical implications for multimedia instruction and learning material design. Educators should conduct emotional design aligned with the emotional valence of learning material—applying positive or negative design to induce learners’ corresponding emotions—thereby enabling students to integrate into cognitive engagement more quickly and efficiently.

### 4.2. The Influence of Emotional Congruency on Eye Movement

To examine how emotional congruency influences learners’ cognitive processes, including selection, organization and integration, this study employed eye-tracking technology for real-time measures during multimedia learning. For positive learning material, the congruent condition exhibited a significantly shorter fixation duration and fewer fixation counts on the text area than the incongruent condition. Similarly, a marginal significant difference also showed in picture area. For negative learning materials, the congruent condition demonstrated a significantly shorter fixation duration and fewer fixation counts on the picture area. According to the Eye-Mind Assumption (Koçoğlu et al., 2021; W. Li et al., 2024; Z. L. Pi et al., 2023), visual fixation reflects the attention allocation of the participants and the difficulty level of processing information. A longer fixation duration and more fixation counts indicate a higher cognitive load. Again, the result of eye movement measures supports the emotional congruency effect. The congruent condition eliminates working memory competition between emotional conflict resolution and semantic processing, enabling faster attentional allocation to learning materials, and, thus, improves the efficiency of learning.

In addition, the congruent condition showed the number of transitions between text and picture AOIs is fewer for both positive and negative materials. According to Stark et al. (2018), increased transitions reflect larger difficulty in organizing and integrating the content in different AOIs. The results further prove that the emotional congruency effect exists through the entire cognitive chain from informational selection and organization to integration, which complements the behavioral performance results. These findings support the study of Navratil et al. (2018), which suggests that a congruent emotional combination promotes the synergistic effect between learners’ emotion and cognition, enhances attentional allocation strategies and optimizes information organization-integration process, thereby reducing participants’ cognitive loads and ultimately results in better learning outcomes.

The eye-tracking measures were highly consistent with post-learning retention and transfer test performance, which indicate that learners’ attentional processes including selection, organization and integration directly impact learning outcomes. This concurrently proves that eye-tracking is an effective online processing measure for multimedia learning research, demonstrating unique advantages in capturing real-time cognitive dynamics (Park et al., 2015). Compared to traditional cognitive assessments, eye-tracking technology provides immediate and objective data during cognitive processing, enabling researchers to simultaneously capture attentional allocation patterns and analyze information processing (Hyönä et al., 2021; W. Li et al., 2024; Z. L. Pi et al., 2023; Rayner, 1998).

### 4.3. The Influence of Emotional Congruency on Subjective Perception

Regarding subjective perceptions, this study found no statistically significant differences between the congruent and incongruent conditions in intrinsic motivation or mental effort, which was contrary to our hypothesis and failed to support the emotional congruency effect. Perceived difficulty was significant in Experiment 1 but not in Experiment 2. Both perceived difficulty and mental effort reflect cognitive load and indicate learners’ cognitive resource allocations and challenge levels during processing (Paas, 1992; Sweller et al., 2011). Intrinsic motivation is defined as interest and enjoyment arising from the learning task itself (Ryan & Deci, 2000), which mirrors learners’ internalization of the emotional valence conveyed by the learning materials (Plass et al., 2014). The post-hoc self-report method was commonly used in previous studies to measure learners’ emotion during learning (Mayer & Estrella, 2014). Notably, many previous studies and meta-analyses showed that emotional design can enhance learners’ motivation (Brom et al., 2018; Mutlu-Bayraktar, 2024; Wong & Adesope, 2021), nevertheless, many related studies about emotional design also reported null effect on motivation and cognitive load (Park et al., 2015; Plass et al., 2014; Um et al., 2012). For example, Fang et al. (2024) conducted a meta-analysis and indicated that positive emotional design failed to significantly change learners’ mental efforts in multimedia learning.

There were several possible explanations for the current null result. The first reason may be that learners may not be sensitive enough to changes in their subjective perception during the learning process (Mayer & Estrella, 2014). Compared with the subjective measurement, eye-tracking results indicated that the congruent condition could motivate learners to engage in more cognitive processing to understand learning content during learning. The second reason may be the limited length of learning; as a potential result of this there may be a weak transfer to the cognitive load and motivation of learners, which was not strong enough to be detected. The third reason may stem from the low intrinsic complexity of the materials. According to cognitive load theory (Chandler, 1991), such subjective perception effects only manifests when tasks impose substantial demands on working memory resources. The fourth reason may be that the current learning task did not activate the high level of interactivity. Previous research showed that the affective impact on cognitive load was amplified when learners must make decisions, solve problems or integrate multiple sources of information (Wang et al., 2023). Finally, according to broaden-and-build theory (Fredrickson, 2001), Plass et al. (2014) suggested that emotional design primarily facilitates learning through positive-emotion-driven deep processing (e.g., integration or transfer), not directly reducing cognitive load or enhancing motivation itself. Future research can adopt multidimensional metrics and advanced psychophysiological tools (e.g., EEG, fNIRS, fMRI) to capture learners’ subjective perceptions.

## 5. Limitations and Future Directions

Firstly, this study was conducted in a laboratory surrounded by university students, who were paid as participants. Consequently, limitations in ecological validity may arise when generalizing findings to other environments or populations. Secondly, the study measured emotion solely through self-report measures; future studies could improve measurement accuracy by combining self-report with physiological methods. Thirdly, the study considered positive and negative emotions; future research may further explore the emotional congruency effects from multiple emotional dimensions, for example, considering the inverted personalization effect (Almeida et al., 2024; Yang et al., 2023), the phenomenon that personalization can actually impair learning effectiveness when studying content that is unpleasant or discomfort inducing. Whether they will also show the emotional congruency effect is a meaningful question. Fourth, the use of anthropomorphic facial expressions and color-coded visuals as emotional design may be effective for the experimental control but are somewhat artificial compared to real educational contexts. These artificial manipulations may not elicit natural emotional responses, thereby limiting the external validity of the study. Fifth, the duration of learning was short and may not adequately affect learners’ emotions; future research may further adopt longer learning materials, which are closer to real learning contexts. Sixth, the present study adopted a visual channel to conduct the emotional design; multimedia learning could be used by the olfactory, gustatory and tactile processing channels, according to the Cognitive-Affective-Social Theory on (digital) Learning Environments (CASTLE; Schneider et al., 2022); social processes triggered by social cues mediate the cognitive processing of information when learning with digital materials so the congruency effect of different information channels could be examined.

## 6. Conclusions

This study employed eye-tracking technology and behavioral methods to investigate the impact of emotional congruency between the emotional valence of learning material and the emotional design on multimedia learning. Firstly, the findings support the emotional mediation hypothesis of the Cognitive-Affective Theory of Multimedia Learning, confirming that learners’ emotions modulate cognitive resource allocation and consequently influence learning performance. Secondly, this research provides empirical evidence to the emotional congruency effect, demonstrating better learning outcomes when the emotional valence of learning material is congruent with the emotional design.

## Figures and Tables

**Table 1:** The emotional states in congruent and incongruent conditions, for both pre-test and post-test.

	Measurement Variables	Congruent *M (SD)*	Incongruent *M (SD)*
Pre-test			
	Positive emotion	30.30 (7.07)	31.15 (6.67)
	Negative emotion	14.24 (4.66)	11.90 (3.99)
Post-test			
	Positive emotion	32.70 (7.92)	27.55 (7.68)
	Negative emotion	12.35 (4.00)	12.25 (3.84)

**Table 2:** The learning outcomes in two experimental conditions.

Measurement Variables	Congruent	Incongruent
	*M (SD)*	*M (SD)*
Retention performance	3.80 (1.06)	3.05 (1.15)
Transfer performance	12.25 (3.78)	9.15 (2.83)

**Table 3:** The results of three subjective perceptions in two conditions.

Measurement Variables	Congruent	Incongruent
	*M (SD)*	*M (SD)*
Intrinsic motivation	5.64 (1.07)	5.26 (1.41)
Perceived difficulty	4.40 (0.35)	5.20 (0.35)
Mental effort	6.00 (1.81)	6.10 (1.17)

**Table 4:** The fixation results on text area and picture area for two experimental conditions.

Dependent Variables	Congruent *M (SD)*	Incongruent *M (SD)*
Fixation duration (ms)		
Text area	11,582 (5952)	14,578 (7944)
Picture area	2750 (2950)	3590 (3234)
Fixation count		
Text area	52.04 (26.76)	66.55 (35.55)
Picture area	11.83 (11.42)	14.97 (11.63)
Number of transitions		
Picture area	3.64 (2.57)	4.81 (3.26)

**Table 5:** The emotional states in congruent and incongruent conditions, for both pre-test and post-test.

	Measurement Variables	Congruent	Incongruent
		*M (SD)*	*M (SD)*
Pre-test			
	Positive emotion	31.75 (6.52)	30.40 (6.11)
	Negative emotion	12.30 (4.78)	15.25 (5.93)
Post-test			
	Positive emotion	26.80 (8.34)	30.10 (4.66)
	Negative emotion	15.55 (9.42)	14.00 (4.41)

**Table 6:** The two learning outcomes in two conditions.

Measurement Variables	Congruent	Incongruent
	*M (SD)*	*M (SD)*
Retention performance	4.10 (1.07)	3.25 (1.61)
Transfer performance	12.75 (4.89)	9.10 (4.84)

**Table 7:** The results of subjective perceptions for two conditions.

Measurement Variables	Congruent	Incongruent
	*M (SD)*	M (*SD*)
Intrinsic motivation	4.63 (1.42)	4.88 (1.07)
Perceived difficulty	5.45 (1.43)	5.35 (1.84)
Mental effort	6.35 (1.60)	6.80 (1.40)

**Table 8:** Fixation results on text area and picture area in two conditions.

Dependent Variables	Congruent *M (SD)*	Incongruent *M (SD)*
Fixation duration (ms)		
Text area	11,496 (7331)	11,806 (7268)
Picture area	1644 (1866)	2391 (2802)
Fixation count		
Text area	53.28 (31.94)	53.96 (31.13)
Picture area	7.07 (8.58)	10.88 (11.91)
Number of transitions		
Picture area	3.98 (2.99)	5.00 (3.38)

## Data Availability

The data that support the findings of this study are available on request from the corresponding author [Y.Z.], upon reasonable request.
